# Genome-Wide Expression Profiling of Genes Associated with the *Lr47*-Mediated Wheat Resistance to Leaf Rust (*Puccinia triticina*)

**DOI:** 10.3390/ijms20184498

**Published:** 2019-09-11

**Authors:** Jiaojiao Wu, Jing Gao, Weishuai Bi, Jiaojie Zhao, Xiumei Yu, Zaifeng Li, Daqun Liu, Bo Liu, Xiaodong Wang

**Affiliations:** 1 College of Plant Protection, Biological Control Center for Plant Diseases and Plant Pests of Hebei, Hebei Agricultural University, Baoding 071000, Hebei, China; wjj15091319740@163.com (J.W.); 18345558629@163.com (J.G.); bws4747@163.com (W.B.); zhaojiaoj918@163.com (J.Z.); lzf7551@aliyun.com (Z.L.); liudaqun@caas.cn (D.L.); 2 State Key Laboratory for Biology of Plant Diseases and Insect Pests, Institute of Plant Protection, Chinese Academy of Agricultural Sciences, No. 2 West Yuanmingyuan Road, Beijing 100193, China; 3 College of Life Sciences, Hebei Agricultural University, Baoding 071000, Hebei, China; yuxiumeizy@126.com; 4 Graduate School of Chinese Academy of Agricultural Sciences, Beijing 100081, China

**Keywords:** Transcriptome, receptor-like kinases, transcription factor, wheat, leaf rust

## Abstract

*Puccinia triticina* (*Pt*), the causal agent of wheat leaf rust, is one of the most destructive fungal pathogens threatening global wheat cultivations. The rational utilization of leaf rust resistance (*Lr*) genes is still the most efficient method for the control of such diseases. The *Lr47* gene introgressed from chromosome 7S of *Aegilops speltoides* still showed high resistance to the majority of *Pt* races collected in China. However, the *Lr47* gene has not been cloned yet, and the regulatory network of the *Lr47*-mediated resistance has not been explored. In the present investigation, transcriptome analysis was applied on RNA samples from three different wheat lines (“Yecora Rojo”, “UC1037”, and “White Yecora”) carrying the *Lr47* gene three days post-inoculation with the epidemic *Pt* race THTT. A comparison between *Pt*-inoculated and water-inoculated “*Lr47*-Yecora Rojo” lines revealed a total number of 863 upregulated (*q*-value < 0.05 and log2foldchange > 1) and 418 downregulated (*q*-value < 0.05 and log2foldchange < −1) genes. Specifically, differentially expressed genes (DEGs) located on chromosomes 7AS, 7BS, and 7DS were identified, ten of which encoded receptor-like kinases (RLKs). The expression patterns of these *RLK* genes were further determined by a time-scale qRT-PCR assay. Moreover, heatmaps for the expression profiles of pathogenesis-related (*PR*) genes and several transcription factor gene families were generated. Using a transcriptomic approach, we initially profiled the transcriptional changes associated with the *Lr47*-mediated resistance. The identified DEGs, particularly those genes encoding RLKs, might serve as valuable genetic resources for the improvement of wheat resistance to *Pt*.

## 1. Introduction

*Puccinia triticina* (*Pt*), the causal agent of wheat leaf rust, is a wide-spread fungal pathogen affecting global wheat production. Wheat leaf rust causes yield losses ranging from 5% to 20%, which can reach approximately 50% during epidemics [1,2]. Although yield losses from leaf rust are normally less damaging than those from stem rust and stripe rust, it causes high level of annual losses due to its more frequent occurrence in more world-wide regions [1,3]. In China, due to the climate change and the large-scale cultivation of less diversified wheat cultivars, the epidemic area of wheat leaf rust has expanded from the Yellow-Huai River Valley area to the major wheat-growing regions [3,4]. In the year of 2012, an epidemic of wheat leaf rust occurred in China on more than 15 million ha and led to an approximately 3 million ton yield losses [5,6]. Currently, the genetic improvement of wheat cultivars using different *Lr* genes is still the most effective solution for this issue.

Until now, worldwide wheat researchers have designated more than 70 *Lr* genes conferring either race-specific resistance or non-race specific adult-plant resistance (APR). Based on the gene-to-gene theory, the race-specific resistance, with a common feature of hypersensitive response (HR), is believed to be controlled by a single dominant *R* gene. Up to now, all the cloned *Lr* genes for the race-specific resistance, including *Lr1*, *Lr10*, and *Lr21*, have been reported to encode receptor-like kinases (RLKs) with nucleotide-binding site and leucine-rich repeat (NBS-LRR) [7,8,9]. On the other hand, APR normally show durable and broad-spectrum resistance to multiple fungal diseases, including yellow rust (*Yr*), leaf rust (*Lr*), stem rust (*Sr*), and powdery mildew (*Pm*). It is time-consuming and labor-intensive for the genetic cloning of quantitative trait loci (QTL) controlling the APR. Currently, only a few APR loci have been cloned in wheat, including *Yr36* encoding a protein kinase with a START domain [10], *Lr34*/*Yr18*/*Pm38* encoding an ABC transporter [11], and *Lr67*/*Yr46* encoding a hexose transporter [12].

Several earlier investigations were conducted to elucidate the transcriptional changes regulated by different *Lr* genes during wheat resistance to *Pt* infection. For example, in one of our previous studies, we identified genes regulated by the *Lr39/41* gene during wheat resistance to *Pt* using suppression subtractive hybridization (SSH) and microarray assays [13]. A recent study compared the transcriptional differences in wheat line carrying the *Lr57* gene between compatible and incompatible interactions [14]. Using a serial analysis of gene expression (SAGE) technique, genes regulated by the *Lr28* gene were initially explored [15]. In the other study, the transcriptional responses in wheat to infections of multiple fungal pathogens, including *Puccinia striiformis* f. sp. *tritici*, *Blumeria graminis* f. sp. *tritici*, and *Fusarium graminearum*, were monitored by a large-scale RNA sequencing approach [16,17]. Transcriptome database was established to understand the APR in a widely-used wheat germplasm “Xingzi 9104” [18].

The previously designated *Lr47* gene was introgressed from chromosome 7S of *Aegilops speltoides* (SS) into chromosome 7AS of *Triticum aestivum* (AABBDD) [19]. A PCR marker was designed based on the 7A allele of *Xabc465*, which was employed in the selection of the *Lr47* gene [20]. Another microsatellite marker *Xgwm60* was discovered to co-segregate with the *Lr47* gene [21]. Agronomic and quality evaluation of different wheat lines carrying the *Lr47* gene was conducted to facilitate wheat breeders in making rational decisions on the application of the *Lr47* gene in their breeding practices [22]. In one of our recent investigations, the *Lr47* gene still showed high resistance to the majority of *Pt* races collected in China [23].

Nevertheless, the *Lr47* gene has not been widely applied in wheat breeding programs in China. This gene has not been cloned yet, and its regulatory network remains largely unknown. In the present investigation, RNA-seq analysis was applied on three different wheat lines (“Yecora Rojo”, “UC1037”, and “White Yecora”) carrying the *Lr47* gene, which were inoculated with the epidemic *Pt* race THTT at three days post-inoculation (dpi), when a clear HR was observed. Transcript reads were assembled using the reference genome of *Triticum aestivum* (AABBDD, “Chinese Spring” TGACv1 version) to determine the DEGs regulated by the *Lr47* gene during the wheat resistance to *Pt* infection. The genome-wide expression patterns of genes associated with the *Lr47*-mediated resistance was initially profiled, which would provide valuable genetic resources for the improvement of wheat resistance to *Pt*.

## 2. Results

### 2.1. Wheat Lines Carrying the Lr47 Gene Showed High Resistance to the Epidemic Leaf Rust Race THTT

**Figure 1:**
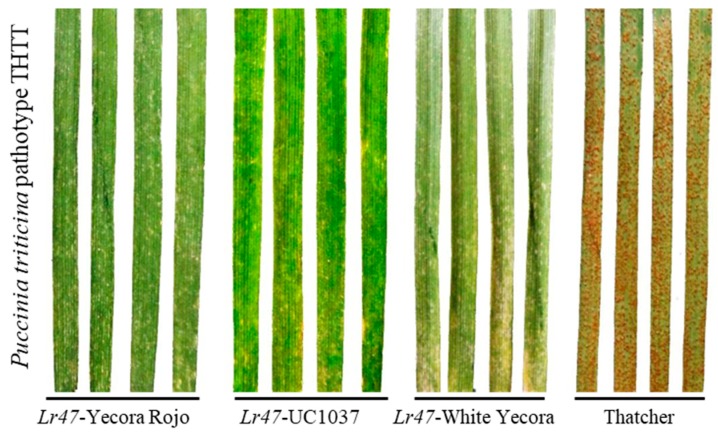
Wheat lines carrying the *Lr47* gene showed high resistance to the prevalent leaf rust race THTT. The seedling plants of three wheat lines carrying the *Lr47* gene in different background of “Yecora Rojo”, “UC1037”, and “White Yecora” were spray-inoculated with water-suspended uredospores of the prevalent leaf rust race THTT. A susceptible line “Thatcher” without any *Lr* genes served as a control. An abundant sporulation of leaf rust was observed in the susceptible line 8 dpi, whereas wheat lines carrying the *Lr47* gene showed necrotic spots phenotype.

In one of our earlier studies, a wheat line introgressed with the *Lr47* gene exerted high resistance to the majority of leaf rust races collected in China, including FHPR, THJS, THTS, FHJQ, PHSL, PHJT, PHSS, KHSS, PHTT, THTT, FHTR, FHHT, TGGT, FHTT, and FGMT [23]. The leaf rust race THTT was predominant in China and showed high virulence to most of the designated *Lr* genes [5]. In the current investigation, we collected wheat lines introgressed with the *Lr47* gene in different spring common wheat cultivars “Yecora Rojo”, “UC1037”, and “White Yecora” from a previous research [23]. The “*Lr47*-Yecora Rojo” was a BC_6_F_5_ line and thus mainly used in the subsequent experiments. Seedling plants of wheat lines “*Lr47*-Yecora Rojo-BC_6_F_5_”, “*Lr47*-UC1037”, and “*Lr47*-White Yecora” were inoculated with water-suspended uredospores of the leaf rust race THTT. A susceptible wheat line “Thatcher” without any *Lr* genes served as a control. Considerable sporulation of *Pt* was observed in the susceptible line 8 dpi, whereas wheat lines carrying the *Lr47* gene showed necrotic spots phenotype (Figure 1).

### 2.2. The Wheat Lines Carrying the Lr47 Gene were Subjected to RNA-Seq Analysis upon Pt Infection

The third leaves of the seedling plants of wheat lines “*Lr47*-Yecora Rojo-BC_6_F_5_”, “*Lr47*-UC1037”, and “*Lr47*-White Yecora” were inoculated with the water-suspended uredospores of leaf rust race THTT. Seedlings of “*Lr47*-Yecora Rojo-BC_6_F_5_” sprayed with water served as a mock control. RNA samples were harvested at 3 dpi from water-inoculated “*Lr47*-Yecora Rojo-BC_6_F_5_”, as well as *Pt*-inoculated “*Lr47*-Yecora Rojo-BC_6_F_5_”, “*Lr47*-UC1037”, and “*Lr47*-White Yecora”. Three biological replicates were collected from each of the inoculated materials. A total number of 12 RNA samples were extracted and subjected to a 12-Gb RNA sequencing approach (Supplementary Table S1). An approximate number of 90–139 million 150-bp pair-end reads were sequenced from each sample and more than 92% of the reads were mapped on the reference genome of *Triticum aestivum* (AABBDD, “Chinese Spring” TGACv1 version, Supplementary Table S2). A total number of 128,794 genes were annotated in the transcriptome, with 14,366 “Novel” transcripts represented contigs that could not be found in the reference genome. For the overall gene expression abundance, we observed clear correlations (*R*^2^ > 0.92) among biological replicates (Supplementary Figure S1). The expression levels of the transcripts in the database were estimated using the fragments per kilobase of transcript per million mapped reads (FPKM) values. The DEGs were identified by DESeq2. All raw data for the transcriptome assembly were deposited in NCBI under BioProject PRJNA498364.

### 2.3. The Overall DEGs Involved in the Lr47-Mediated Resistance

From the comparison made between *Pt*-inoculated “*Lr47*-Yecora Rojo-BC_6_F_5_” and water-inoculated “*Lr47*-Yecora Rojo-BC_6_F_5_”, a total number of 863 genes were significantly upregulated (*q*-value < 0.05, log2foldchange > 1) during the *Lr47*-mediated resistance, whereas 418 genes were significantly downregulated (*q*-value < 0.05, log2foldchange < −1). We observed a broad range of transcripts encoding RLKs and Cytochrome P450 in the identified upregulated DEGs, whereas several heat-stress-responsive genes were significantly suppressed during the *Lr47*-mediated resistance.

**Figure 2:**
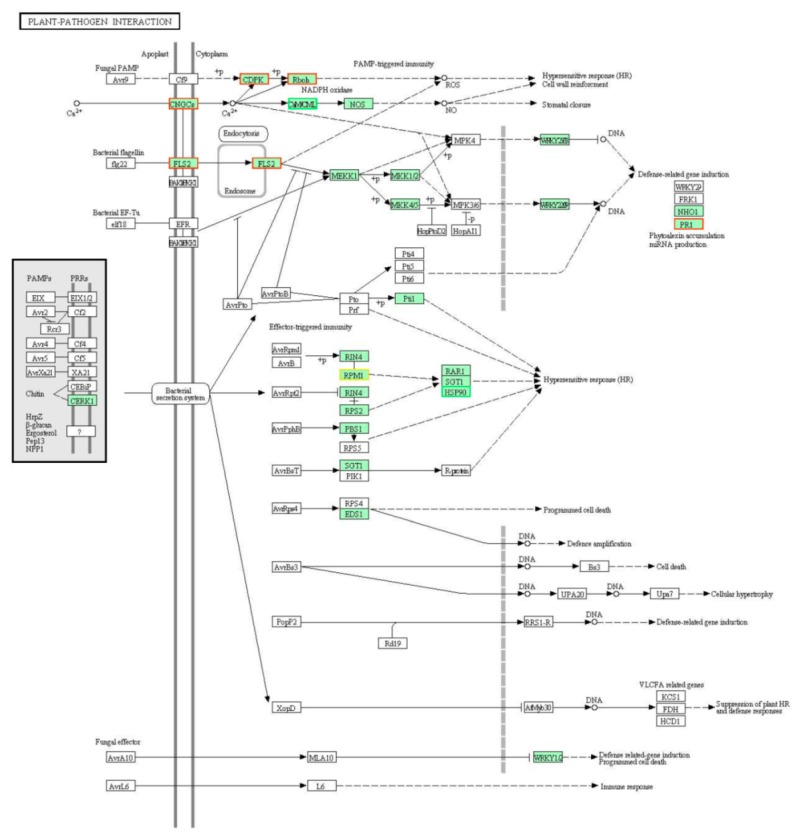
The “plant–pathogen interaction” KEGG pathway annotations for the identified DEGs. Nodes of KEGG pathway enriched with DEGs were colored in green. Detailed information for the significant upregulated (frame in red color) or downregulated (frame in bright green color) DEGs was presented in Table 1.

Based on the GO annotations, the overall information for either upregulated or downregulated DEGs was summarized in three main categories of “molecular function”, “cellular components”, and “biological process” (Supplementary Figure S2). For the molecular function, the majority of both upregulated and downregulated DEGs were significantly associated with “binding” and “catalytic activity”, whereas a portion of genes enriched in “response to stimulus” in the upregulated DEGs was higher than that in the downregulated ones. For the Kyoto Encyclopedia of Genes and Genomes (KEGG) pathway analysis, several DEGs, including Ca^2+^ pathway genes (*CNGCs*, *CDPK*, *Rboh*, and *CaM*), *FLS2*, *RPM1*, *PR1*, and *HSP90*, were annotated in the “plant–pathogen interaction” pathway (Figure 2 and Table 1).

### 2.4. Transcriptional-Induced Genes Located on Chromosomes 7AS, 7BS, and 7DS were Identified

**Figure 3:**
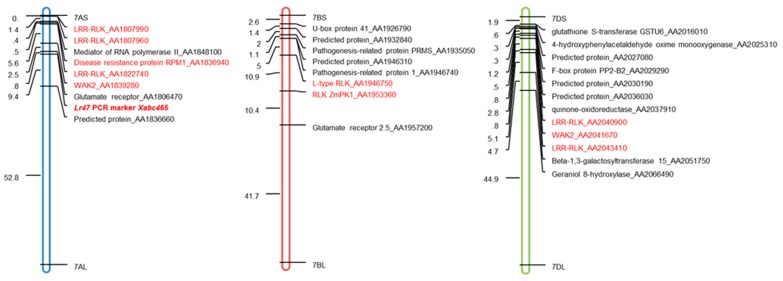
Distribution map of transcriptional-induced genes located on chromosomes 7AS, 7BS, and 7DS. The race-specific resistance gene *Lr47* was introgressed from chromosome 7S of *Aegilops speltoides* to chromosome 7AS of hexaploid wheat *Triticum aestivum*. Previous genetic study identified a PCR marker *Xabc465* that co-segregated with the *Lr47* gene. A total number of 28 upregulated genes located on chromosomes 7AS, 7BS, and 7DS were identified from the assembled transcriptome, and ten of which encoded RLKs (labeled in red color, detailed information in Table 2). The physical position for each of the gene was determined and anchored to the distribution map using MapMaker software.

Since the chromosome segment carrying the *Lr47* gene was introgressed from chromosome 7S of *Aegilops speltoides* to the chromosome 7AS of common wheat [19], we focused on the exploration of transcriptional-induced genes located on chromosomes 7AS, 7BS, and 7DS. Significantly upregulated (*q*-value < 0.05 and log2foldchange > 1) in comparison between *Pt*-inoculated “*Lr47*-Yecora Rojo-BC_6_F_5_” and water-inoculated “*Lr47*-Yecora Rojo-BC_6_F_5_”, a total number of 28 genes located on chromosomes 7AS, 7BS, and 7DS were identified. The chromosome distributions for all such genes were anchored based on their physical positions (Figure 3). Interestingly, ten of them encoded different types of RLKs, including Leucine-rich repeat receptor-like serine/threonine-protein kinase (LRR-RLK), Disease resistance protein RPM1, Wall-associated receptor-like kinase (WAK), and L-type RLK (Table 2). The closest homologs of these RLKs in other plant species including *Aegilops tauchii* (Att), *Oryza sativa* (Os), and *Brachypodium distachyon* (Bd), as well as the proteins encoded by *Lr1*, *Lr10*, *Lr21*, *Lr34*, and *Lr67* genes, were employed to generate a neighbor-joining tree (Supplementary Figure S3). From the polygenetic analysis, we noticed that LRR-RLK_AA1822740 (7AS) showed high similarity with LRR-RLK_AA2040900 (7DS), and WAK2_AA1839280 (7AS) was a homolog of WAK2_AA2041670 (7DS).

**Figure 4:**
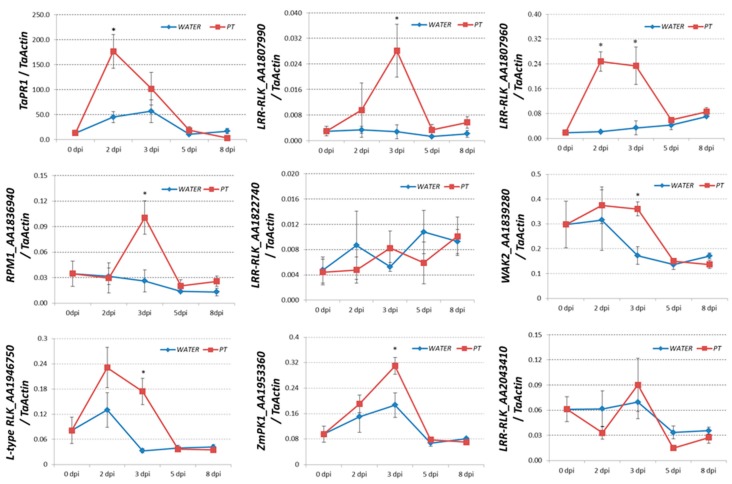
The expression profiles of selected genes encoding RLKs during the *Lr47*-mediated resistance were determined by qRT-PCR assay. Fully-expanded third leaves of “*Lr47*-Yecora Rojo BC_6_F_5_” line were spray-inoculated with leaf rust race THTT. Seedling plants of the same material sprayed with water served as a control. RNA samples were harvested at 0, 2, 3, 5, and 8 dpi. Five independent biological replicates were included. The transcript levels of all genes were expressed as linearized fold-*TaActin* levels using the 2^−ΔCt^ method. Wheat pathogenesis-related *TaPR1* gene was employed as a positive control. Mean and standard error of relative expressions were calculated, and two-sample *t*-test (*P < 0.05) was conducted using Microsoft Excel software.

To validate the expression profiles of these chromosome 7AS/7BS/7DS-located upregulated *RLK* genes, a time-scale qRT-PCR assay was performed. Fully-expanded third leaves of the “*Lr47*-Yecora Rojo-BC_6_F_5_” line were spray-inoculated with uredospores of the leaf rust race THTT. RNA samples were harvested at 0, 2, 3, 5, and 8 dpi. Seedling plants of the “*Lr47*-Yecora Rojo-BC_6_F_5_” line sprayed with water served as a control. The expression level of a wheat gene encoding pathogenesis-related protein 1 (*TaPR1*, GenBank accession FJ815169.1) was significantly (*P < 0.05) upregulated at 2 dpi during the *Lr47*-mediated resistance (Figure 4), indicating that a regular plant defense response was triggered. The expression levels of genes encoding LRR-RLK_AA1807990, LRR-RLK_AA1807960, RPM1_AA1836940, WAK2_AA1839280, L-type RLK_AA1946750, and ZmPK1_AA1953360, were significantly (* *P* < 0.05) induced at 3 dpi. On the other hand, we did not observe any significant inductions of genes encoding LRR-RLK_AA1822740 and LRR-RLK_AA2043410.

### 2.5. The Expression Patterns of Genes Encoding PR Proteins and Several Transcription Factor Families during the Lr47-Mediated Resistance were Profiled

**Figure 5:**
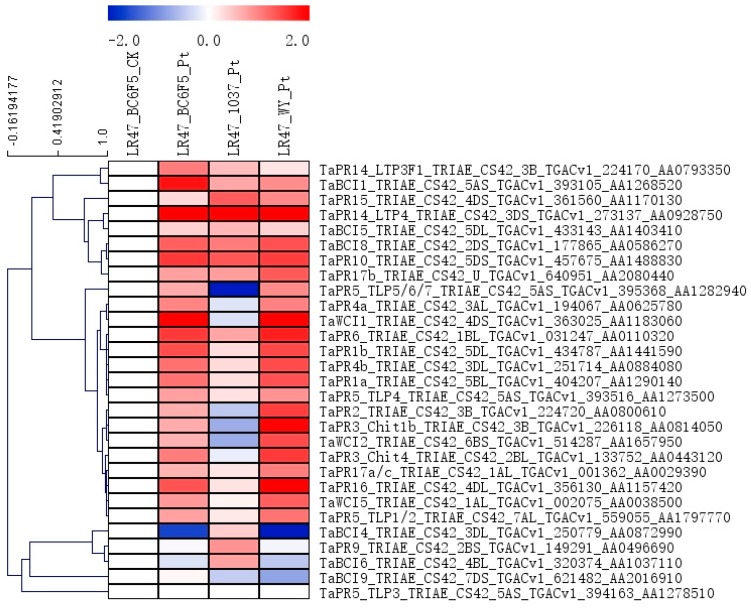
The expression profiles of pathogenesis-related (*PR)* and BTH-sensitive genes during the *Lr47*-mediated resistance. FPKM values for each of the genes in “LR47_BC6F5_Pt”, “LR47_1037_Pt”, and “LR47_WY_Pt” were relative to that in “LR47_BC6F5_CK”. A log2foldchange data transformation was conducted using Microsoft Excel software. A heatmap was generated by MeV software using the relative expression data of *PR* and BTH-sensitive (*WCI* and wheat homologs of *BCI*) genes. Genes with similar expression patterns were clustered using the “hierarchical clustering” function of the MeV software. LR47_BC6F5_CK: water-inoculated “Lr47-Yecora Rojo-BC_6_F_5_”, LR47_BC6F5_Pt: *Pt*-inoculated “Lr47-Yecora Rojo-BC_6_F_5_”, LR47_1037_Pt: *Pt*-inoculated “Lr47-UC1037”, LR47_WY_Pt: *Pt*-inoculated “Lr47-White Yecora”.

To determine the possible downstream genes and key regulators of the *Lr47*-mediated resistance, the expression patterns of the genes encoding PR proteins and several transcription factor families were profiled using their FPKM values in the transcriptome database. Until the time of conducting our investigation, a total number of 18 gene families had been named as *PR* genes from various plant species in response to pathogen infections, but only a few of them had been cloned or characterized in *Triticeae* crops of wheat and barley [24]. In the present investigation, we found that most of the *PR* genes excluding *TaPR9* were significantly upregulated during the *Lr47*-mediated resistance at 3 dpi (Figure 5). Wheat chemical-induced (*WCI*) genes and barley chemical-induced (*BCI*) genes have been reported to be sensitive to the treatment of benzothiadiazole (BTH, a salicylic acid homolog) [25,26]. Surprisingly, the majority of the *WCI* genes and homologs of *BCI* genes, excluding *BCI4*, *BCI6*, and *BCI9*, also showed significant inductions upon *Pt* infection in wheat lines carrying the *Lr47* gene (Figure 5).

**Figure 6:**
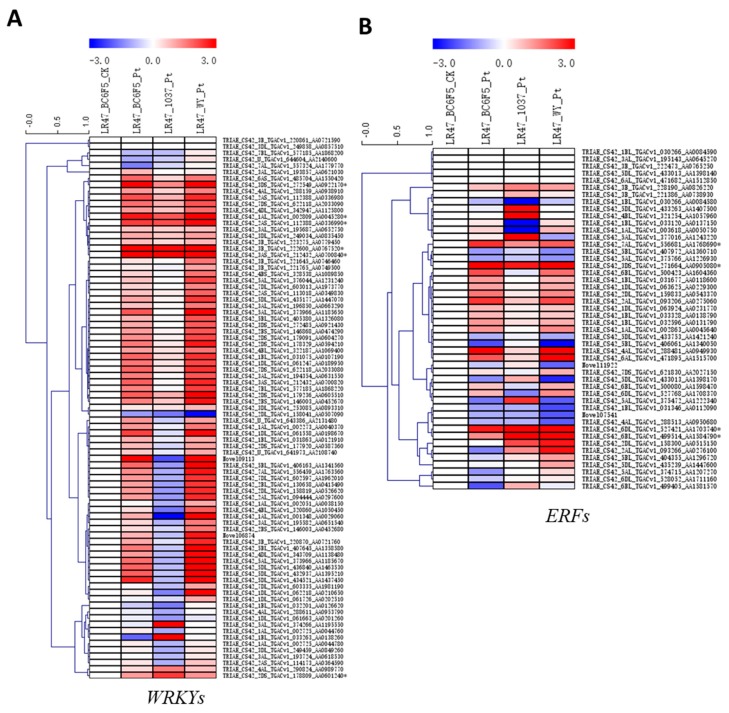
The expression profiles of the genes encoding (**A**) WRKY and (**B**) ERF transcription factors during the *Lr47*-mediated resistance. FPKM values for each of the genes in “LR47_BC6F5_Pt”, “LR47_1037_Pt”, and “LR47_WY_Pt” were relative to that in “LR47_BC6F5_CK”. A log2foldchange data transformation was conducted using Microsoft Excel software. Heatmaps were generated by MeV software using the relative expression data of the genes encoding the WRKY and ERF transcription factors, respectively. Genes with similar expression patterns were clustered using the “hierarchical clustering” function of the MeV software. Constantly induced genes were labeled with asterisk (*), and the deduced proteins of which were employed to generate polygenetic trees in Supplementary Figures S5 and S6, respectively. LR47_BC6F5_CK: water-inoculated “Lr47-Yecora Rojo-BC_6_F_5_”, LR47_BC6F5_Pt: *Pt*-inoculated “Lr47-Yecora Rojo-BC_6_F_5_”, LR47_1037_Pt: *Pt*-inoculated “Lr47-UC1037”, LR47_WY_Pt: *Pt*-inoculated “Lr47-White Yecora”.

The expression profiles of several gene families encoding transcription factors, including WRKY, ERF, MYB, bHLH, bZIP, and NAC, were generated using their FPKM values, respectively. Interestingly, large portions of *WRKY* and *ERF* genes were significantly upregulated during the *Lr47*-mediated resistance (Figure 6), whereas only a few members of *MYB, bHLH*, and *bZIP* genes showed constant inductions among wheat lines carrying the *Lr47* gene in different genetic backgrounds (Supplementary Figure S4). Several polygenetic analyses were conducted using transcription factors encoded by the selected differentially expressed *WRKY*, *ERF*, *MYB*, *bHLH*, and *bZIP* genes and their closest homologs in other plant species, respectively (Supplementary Figures S5–S7).

## 3. Discussion

In the present investigation, the transcriptional changes associated with the *Lr47*-mediated wheat resistance to leaf rust were initially profiled. Approximately, only one-third of the designated *Lr* resistance loci are effective against the prevalent *Pt* race THTT in China [23]. The wheat isogenic line introgressed with the *Lr47* gene still showed high resistance to the majority of leaf rust races collected in China [27]. The seedling resistance gene *Lr47* was introgressed from chromosome 7S of *Aegilops speltoides* to chromosome 7AS of common wheat [19]. However, this gene has not been widely-used in wheat breeding programs in China. In the present investigation, wheat lines carrying the *Lr47* gene in different genetic backgrounds (“Yecora Rojo”, “UC1037”, and “White Yecora”) may serve as valuable germplasm resources (Figure 1). Despite their excellent resistance to leaf rust, wheat lines carrying the *Lr47* gene had clear drawbacks in terms of grain yield (overall reduction by 3.8%, 220 kg/ha) and flour yield (21.8 g/kg), possibly due to the introgression of other genes co-segregated with *Lr47* from *Aegilops speltoides* [22]. Consequently, gene cloning and mechanism exploration of the *Lr47* gene will considerably facilitate the further practical application of this gene.

Due to the large (17 Gb) and polyploid (AABBDD) genome of common wheat, the identification of defense-related genes upon pathogen infection has never been an easy task. A total number of 863 upregulated and 418 downregulated DEGs were annotated in the transcriptome database. Clear activations of genes encoding RLKs were detected, some of which may be involved as key sensors or signal transducers during the HR conferred by the *Lr47* gene. In an earlier investigation, a large amount of genes encoding RLKs were transcriptional induced upon infections of wheat stripe rust and powdery mildew [16]. Several genes encoding RLKs were upregulated in rice inoculated with non-host pathogen wheat leaf rust [28]. Interestingly, we also detected higher accumulation of genes encoding Cytochrome P450, metabolic products of which may be critically involved in the plant defense against insects and pathogens [29]. For the downregulated DEGs, we found that several heat-stress-responsive genes were significantly suppressed, including genes encoding heat-shock protein, heat-stress transcription factor, and the abscisic acid (ABA) receptor PYL4. The signaling hormone ABA was reported to be accumulated upon heat stress [30], whereas it negatively affected disease resistance by interfering with other phytohormones, such as salicylic acid, jasmonic acid, and ethylene [31]. Based on the suppression of multiple ABA-responsive genes established in our transcriptome database, we speculated that ABA exerted a negative role during the *Lr47*-mediated resistance.

For the enrichment of DEGs in the KEGG pathway of “plant–pathogen interaction”, we found a clear involvement of the calcium (Ca^2+^) signal pathway in the *Lr47*-mediated resistance (Figure 2 and Table 1). Several genes in this pathway, including those encoding calcineurin B-like proteins (CBLs), CBL-interacting protein kinases (CIPKs), and cyclic nucleotide gated channels (CNGCs), were previously reported to participate in the wheat resistance to stripe rust [32,33].

Currently, all the cloned race-specific *Lr* genes (*Lr1*, *Lr10*, and *Lr21*) in wheat encoded NBS-LRR RLKs [7,8,9]. Approximately, 3400 full-length NBS-LRR RLK loci have been identified in common wheat, 1540 of which have been further confirmed as complete genes [34]. RLKs play crucial roles in both pathogen recognition and signal transduction during plant-pathogen interactions [35]. They may act through direct detection of the presence of pathogen effector molecules [36], but, more frequently, they exert their functions indirectly through sensing the host target modification by the pathogen effector [37].

In the current study, a total number of ten chromosome 7AS/7BS/7DS-located upregulated *RLK* genes were identified (Table 2). The physical positions of such genes, as well as the linkage marker *Xabc465* for *Lr47* introgression, were predicted based on the blast results in the common wheat reference genome (Figure 3). Most of the identified *RLK* genes were significantly upregulated during the *Lr47*-mediated resistance in our subsequent qRT-PCR validation assay (Figure 4), the functions of which require further exploration.

We further extracted the expression data of several gene families that may be functioning during wheat resistance to leaf rust. For instance, a total number of 18 gene families were named as *PR* genes from various plant species in response to pathogen infections, but only a few of them were cloned or characterized in wheat [24]. Another group of genes, including *WCI* and *BCI* genes, was previously reported to be sensitive to the BTH treatment [25,26]. In the present investigation, most of the *PR*, *WCI*, and homologs of *BCI* genes were significantly upregulated upon *Pt* infection during the *Lr47*-mediated resistance (Figure 5), indicating that a wide range of salicylic acid-related plant defenses were recruited by the *Lr47* gene.

Transcription factors are key components of the gene regulatory networks in various biological processes. Several transcription factor gene families, including *WRKY*, *ERF*, *MYB*, *bHLH*, *bZIP*, and *NAC*, have been reported to play crucial roles during plant defense and stress responses [38,39,40,41]. In the present investigation, we detected more pronounced inductions in the *WRKY* and *ERF* transcription factors during the *Lr47*-mediated resistance (Figure 6). The naming system for transcription factors in common wheat or its relative species was confused. Our polygenetic analyses for the selected transcription factors provided initial clues to explore the key nodes of the *Lr47*-mediated resistance (Supplementary Figures S5–S7).

Wheat lines carrying the *Lr47* gene showed great potential in improving wheat resistance to *Pt* infection in China. In the current study, we initially profiled the regulatory network of the *Lr47*-mediated resistance. A total of ten chromosome 7AS/7BS/7DS-located upregulated *RLK* genes were identified, and their expression patterns during the *Lr47*-mediated resistance were further determined by qRT-PCR assay. Specific *PR* genes, as well as those differentially expressed transcription factor genes, might be valuable genetic resources for the improvement of wheat resistance to *Pt*. The transcriptome itself will greatly facilitate the cloning process of the *Lr47* gene.

## 4. Materials and Methods

### 4.1. Plants Growth and Leaf Rust Inoculation

Wheat lines introgressed with the *Lr47* gene in different genetic backgrounds of “Yecora Rojo”, “UC1037”, and “White Yecora” were derived from previous study [22] and collected from Prof. Jorge Dubcovsky’s lab at University of California, Davis. The epidemic *Pt* race THTT was isolated from the field as described in one of our former studies [23] and preserved in our lab. Wheat seedlings were kept in the greenhouse and inoculated with leaf rust as described [41]. The third leaves of the seedlings were sprayed with water-suspended uredospores of *Pt* race THTT upon their full expansion. Seedlings sprayed with water served as a mock control. Phenotype of the leaf rust was photographed at 10 dpi. RNA samples for Illumina sequencing were harvested at 3 dpi. For qRT-PCR assay, inoculated leaves were sampled from the “*Lr47*-Yecora Rojo-BC_6_F_5_” line at 0, 2, 3, 5, and 8 dpi. We set four independent biological replicates for each time point. All samples were rapidly frozen in liquid nitrogen and then ground into a fine powder.

### 4.2. RNA-seq and Bioinformatics Analysis

Total RNA for each of the samples was extracted using an RNA extraction kit (Qiagen, Hilden, Germany) following the manufacturer’s instructions. Library preparation and sequencing procedure were conducted by Novogene Co., Ltd. following the KAPA Library preparation and Illumina HiSeq protocols (Illumina, San Diego, CA, USA). The RNA sequencing was run on a HiSeq 1000 instrument. A quality analysis was conducted to remove adapters and overrepresented sequences using in-house perl scripts. Raw reads with adapters, N ratio higher than 10%, or ratio of poor base (Q_phred_ ≤ 20) higher than 50%, were removed. Clean reads were mapped on the reference genome of *Triticum aestivum* (AABBDD, “Chinese Spring” TGACv1 version) [42] using TopHat 2.0.8 software with default parameter settings for 150-bp pair-end sequencing reads [43]. Multi-mapped reads and low mapping reads were identified using hisat2 software, and reads with MAPQ = 60 were designated as high qualified uniq reads. Transcripts were extracted using Cufflinks v2.1.1 software with default parameter. All transcripts were compared with gene models in the reference genome using Cuffcompare, and those assembled contigs that could not be found in the reference genome (class_code “u”) were annotated as “Novel” transcripts. Then, the expression levels of genes were determined using HTSeq v0.9.1 software [44]. Briefly, raw_readcount from HTSeq was employed to calculate the expected number of fragments per kilobase of transcript sequence per Millions base pairs (FPKM) sequenced, which have considered the effect of sequencing depth and gene length for the reads count at the same time, and is currently the most commonly used method for estimating gene expression levels [44]. DEGs were isolated by filtering the expression levels of genes between *Pt*-inoculated “*Lr47*-Yecora Rojo-BC_6_F_5_” and water-inoculated “*Lr47*-Yecora Rojo-BC_6_F_5_” for an FDR-adjusted *p* value < 0.05 using the DESeq2 software [45]. The GOseq package software was employed to give each of the genes in the transcriptome database a GO annotation [46]. Summaries of GO annotation categories for the selected genes were generated using function of “GOLevel2 Counter” in TBtools software. Using FPKM values for the selected genes from the transcriptome database, several heatmaps were generated by MeV software. Gene clusters were divided based on the expression patterns using the “hierarchical clustering” function of the MeV software following the “Pearson correlation” distance metric and “average linkage clustering” linkage method. To characterize the complex biological behaviors of the *Lr47*-mediated resistance, KEGG annotations were utilized to analyze the regulatory pathways enriched with DEGs. The physical positions for DEGs located on chromosomes 7AS, 7BS, and 7DS were collected from the high qualified reference genome of chromosome 7 [47]. The distribution map was generated using MapMaker software version 3.0. Multiple sequence alignments were conducted using the MUSCLE method and neighbor-joining trees were generated through the MEGA software version 7.0. The confidence of nodes in each of the neighbor-joining tree were calculated using 1000 bootstrap cycles.

### 4.3. qRT-PCR Assay

RNAs from leaf samples were isolated using a Qiagen RNA extraction kit. A Takara Reverse Transcription Kit was employed for the synthesis of the first-strand cDNA. Equal amount of the total RNA for each of the sample was used to synthesize cDNA. qRT-PCR primers for each of the tested genes were designed (Supplementary Table S3). The wheat *TaActin* gene (GenBank accession AB181991.1) was employed as an inner reference gene [48]. The amplification efficiency was determined by a preliminary qRT-PCR assay using six 2-fold diluted cDNA samples (1:1, 1:2, 1:4, 1:8, 1:16, and 1:32). qRT-PCR reactions were performed using TransGen SYBR Green qPCR mix with a Bio-Rad CFX Manager instrument under the following conditions: 3 min at 95 °C; 40 cycles of 10 s at 95 °C, 10 s at 60 °C, and 10 s at 72 °C. Melting curves ranging from 60 °C to 94 °C were recorded by the instrument to evaluate the amplified product. The expression levels of target genes were relative to that of the reference *TaActin* gene in the same sample using the 2^-ΔCt^ method [49,50]. The relative expression represents the ratio between the initial number of molecules of the target gene and that of *TaActin*. Therefore, the Y scales of the qRT-PCR results are comparable across genes and experiments.

## Figures and Tables

**Table 1:** DEGs annotated in the “plant–pathogen interaction” KEGG pathway.

KEGG Annotation	Gene ID	R47_CK FPKM	R47_PT FPKM	R47_1037 FPKM	R47_WY FPKM	R47_PT vs R47_CK *P* Adjust	Gene Annotation
CNGCs	TRIAE_CS42_4BL_TGACv1_322377_AA1070960	6.93	10.86	0.85	0.66	1.1 × 10^−5^	Zinc finger RING/FYVE/PHD-type protein
CDPK	TRIAE_CS42_5AL_TGACv1_374737_AA1207890	0.18	0.80	8.13	10.42	2.0 × 10^−2^	EF-Hand calcium-binding protein
	TRIAE_CS42_2AS_TGACv1_112475_AA0338720	195.08	99.02	7.65	7.89	3.2 × 10^−2^	TRIAE calcium-binding protein
Rboh	TRIAE_CS42_3AL_TGACv1_195736_AA0653240	0.45	3.18	0.16	0.37	6.0 × 10^−4^	Respiratory burst oxidase homologs
CaM	TRIAE_CS42_5AL_TGACv1_375077_AA1215670	0.98	0.45	2.18	1.52	2.5 × 10^−2^	TRIAE calcium-binding protein
	TRIAE_CS42_2BS_TGACv1_146898_AA0475060	25.78	13.30	4.35	1.66	3.8 × 10^−3^	TRIAE calcium-binding protein
	TRIAE_CS42_1DS_TGACv1_080174_AA0242230	184.29	92.24	7.04	6.37	3.0 × 10^−2^	TRIAE calcium-binding protein
	TRIAE_CS42_1BS_TGACv1_050118_AA0167620	0.17	0.98	4.70	2.70	4.7 × 10^−2^	TRIAE calcium-binding protein
FLS2	TRIAE_CS42_2AL_TGACv1_094921_AA0304880	0.59	2.02	0.34	0.62	5.0 × 10^−4^	LRR receptor-like serine/threonine-protein kinase FLS2
RPM1	TRIAE_CS42_7AS_TGACv1_570514_AA1836940	1.02	1.73	0.67	1.16	2.5 × 10^−4^	NB-ARC, P-loop containing nucleoside triphosphate hydrolase
	TRIAE_CS42_4AL_TGACv1_290009_AA0980370	4.04	1.83	0.49	0.35	3.2 × 10^−2^	NB-ARC, P-loop containing nucleoside triphosphate hydrolase
	TRIAE_CS42_2AS_TGACv1_114436_AA0367230	0.47	1.08	0.09	0.41	2.7 × 10^−2^	NB-ARC, P-loop containing nucleoside triphosphate hydrolase
	TRIAE_CS42_1DS_TGACv1_080256_AA0244390	5.26	13.59	1.22	2.83	3.3 × 10^−2^	NB-ARC, P-loop containing nucleoside triphosphate hydrolase
PR1	TRIAE_CS42_U_TGACv1_709776_AA2166170	5.52	2.89	3.06	0.87	1.9 × 10^−2^	Cysteine-rich secretory protein
	TRIAE_CS42_7BS_TGACv1_592960_AA1946740	1.52	2.46	5.40	8.16	4.5 × 10^−2^	Cysteine-rich secretory protein
	TRIAE_CS42_7BS_TGACv1_592288_AA1935050	0.26	0.69	1.75	1.96	4.1 × 10^−2^	Cysteine-rich secretory protein
	TRIAE_CS42_5BL_TGACv1_405770_AA1335010	0.74	0.25	22.06	37.50	3.1 × 10^−2^	Cysteine-rich secretory protein
	TRIAE_CS42_5BL_TGACv1_405157_AA1321310	0.13	0.39	3.83	3.28	8.1 × 10^−14^	Cysteine-rich secretory protein
HSP90	TRIAE_CS42_7BS_TGACv1_593752_AA1954160	10.16	5.17	97.98	135.18	7.4 × 10^−3^	Heat shock protein Hsp90
	TRIAE_CS42_7AS_TGACv1_571863_AA1850400	6.02	9.48	105.27	154.30	1.1 × 10^−2^	Heat shock protein Hsp90
	TRIAE_CS42_5BS_TGACv1_423847_AA1384070	5.20	2.04	14.29	14.90	1.6 × 10^−2^	Heat shock protein Hsp90
	TRIAE_CS42_2DS_TGACv1_177184_AA0568130	24.49	61.73	0.81	0.31	8.9 × 10^−3^	Heat shock protein Hsp90

R47_CK: water-inoculated “*Lr47*-Yecora Rojo-BC_6_F_5_”, R47_PT: *Pt*-inoculated “*Lr47*-Yecora Rojo-BC_6_F_5_”, R47_1037: *Pt*-inoculated “*Lr47*-UC1037”, R47_WY: *Pt*-inoculated “*Lr47*-White Yecora”.

**Table 2:** Transcriptional-induced genes encoding RLKs located on chromosomes 7AS, 7BS, and 7DS.

Gene ID	R47_CK FPKM Value	R47_PT FPKM Value	R47_1037 FPKM Value	R47_WY FPKM Value	R47_PT vs R47_CK *P* Adjust Value	Gene Annotation
TRIAE_CS42_7AS_TGACv1_569126_AA1807990	1.44	3.37	1.94	3.63	8.4 × 10^−^^4^	Putative LRR receptor-like serine/threonine-protein kinase
TRIAE_CS42_7AS_TGACv1_569126_AA1807960	0.44	1.33	0.78	1.31	5.7 × 10^−^^3^	Putative LRR receptor-like serine/threonine-protein kinase
TRIAE_CS42_7AS_TGACv1_570514_AA1836940	0.47	1.08	0.67	1.16	2.5 × 10^−^^4^	Putative LRR receptor-like serine/threonine-protein kinase
TRIAE_CS42_7AS_TGACv1_569722_AA1822740	0.09	0.81	0.16	0.40	7.2 × 10^−^^5^	Putative LRR receptor-like serine/threonine-protein kinase
TRIAE_CS42_7AS_TGACv1_570678_AA1839280	0.17	0.50	0.37	0.74	9.15 × 10^−^^6^	Wall-associated receptor kinase 2
TRIAE_CS42_7BS_TGACv1_592960_AA1946750	3.39	7.68	5.04	8.88	5.91 × 10^−^^4^	L-type receptor-like protein kinase IX.1
TRIAE_CS42_7BS_TGACv1_593644_AA1953360	1.25	2.54	2.87	3.11	1.86 × 10^−^^5^	Receptor-like protein kinase ZmPK1
TRIAE_CS42_7DS_TGACv1_622500_AA2040900	0.00	0.11	0.04	0.03	5.84 × 10^−^^5^	Putative LRR receptor-like serine/threonine-protein kinase
TRIAE_CS42_7DS_TGACv1_622551_AA2041670	0.77	1.58	1.18	1.85	2.4 × 10^−^^2^	Wall-associated receptor kinase 2
TRIAE_CS42_7DS_TGACv1_622658_AA2043410	0.71	1.96	2.83	1.82	1.8 × 10^−^^4^	Putative LRR receptor-like serine/threonine-protein kinase

R47_CK: water-inoculated “*Lr47*-Yecora Rojo-BC_6_F_5_”, R47_PT: *Pt*-inoculated “*Lr47*-Yecora Rojo-BC_6_F_5_”, R47_1037: *Pt*-inoculated “*Lr47*-UC1037”, R47_WY: *Pt*-inoculated “*Lr47*-White Yecora”.

## Supplementary Materials

The following are available online at https://www.mdpi.com/1422-0067/20/18/4498/s1.

## References

[1] Bolton M.D., Kolmer J.A., Garvin D.F. (2008). Wheat leaf rust caused by Puccinia triticina. Mol. Plant Pathol..

[2] Eversmeyer M.G., Kramer C.L. (2000). Epidemiology of wheat leaf and stem rust in the central great plains of the USA. Annu. Rev. Phytopathol..

[3] Huerta-Espino J., Singh R., German S., McCallum B., Park R., Chen W.Q., Bhardwaj S., Goyeau H. (2011). Global status of wheat leaf rust caused by Puccinia triticina. Euphytica.

[4] Helfer S. (2014). Rust fungi and global change. New Phytol..

[5] Zhang L., Meng Q., Yan H., Liu D. (2019). Virulence and molecular genetic diversity of the Puccinia triticina population in Hebei Province of China in 2008 and 2010. Eur. J. Plant Pathol..

[6] Zhou H., Xia X., He Z., Li X., Wang C., Li Z., Liu D. (2013). Molecular mapping of leaf rust resistance gene LrNJ97 in Chinese wheat line Neijiang 977671. Theor. Appl. Genet..

[7] Cloutier S., McCallum B.D., Loutre C., Banks T.W., Wicker T., Feuillet C., Keller B., Jordan M.C. (2007). Leaf rust resistance gene Lr1, isolated from bread wheat (Triticum aestivum L.) is a member of the large psr567 gene family. Plant Mol. Biol..

[8] Feuillet C., Travella S., Stein N., Albar L., Nublat A., Keller B. (2003). Map-based isolation of the leaf rust disease resistance gene Lr10 from the hexaploid wheat (Triticum aestivum L.) genome. Proc. Natl. Acad. Sci. USA.

[9] Huang L., Brooks S.A., Li W., Fellers J.P., Trick H.N., Gill B.S. (2003). Map-based cloning of leaf rust resistance gene Lr21 from the large and polyploid genome of bread wheat. Genetics.

[10] Fu D., Uauy C., Distelfeld A., Blechl A., Epstein L., Chen X., Sela H., Fahima T., Dubcovsky J. (2009). A kinase-START gene confers temperature-dependent resistance to wheat stripe rust. Science.

[11] Krattinger S.G., Lagudah E.S., Spielmeyer W., Singh R.P., Huerta-Espino J., McFadden H., Bossolini E., Selter L.L., Keller B. (2009). A putative ABC transporter confers durable resistance to multiple fungal pathogens in wheat. Science.

[12] Moore J.W., Herrera-Foessel S., Lan C., Schnippenkoetter W., Ayliffe M., Huerta-Espino J., Lillemo M., Viccars L., Milne R., Periyannan S. (2015). A recently evolved hexose transporter variant confers resistance to multiple pathogens in wheat. Nat. Genet..

[13] Li X., Wang X., Kang Z., Ren Z., Bi W., Yang W., Liu D. (2018). Suppression subtractive hybridization and microarray analysis reveal differentially expressed genes in the Lr39/41-mediated wheat resistance to Puccinia triticina. Eur. J. Plant Pathol..

[14] Yadav I.S., Sharma A., Kaur S., Nahar N., Bhardwaj S.C., Sharma T.R., Chhuneja P. (2016). Comparative temporal transcriptome profiling of wheat near isogenic line carrying Lr57 under compatible and incompatible interactions. Front. Plant Sci..

[15] Singh D., Kumar D., Satapathy L., Pathak J., Chandra S., Riaz A., Bhaganagre G., Dhariwal R., Kumar M., Prabhu K.V. (2017). Insights of Lr28 mediated wheat leaf rust resistance: Transcriptomic approach. Gene.

[16] Zhang H., Yang Y., Wang C., Liu M., Li H., Fu Y., Wang Y., Nie Y., Liu X., Ji W. (2014). Large-scale transcriptome comparison reveals distinct gene activations in wheat responding to stripe rust and powdery mildew. BMC Genom..

[17] Xiao J., Jin X., Jia X., Wang H., Cao A., Zhao W., Pei H., Xue Z., He L., Chen Q. (2013). Transcriptome-based discovery of pathways and genes related to resistance against Fusarium head blight in wheat landrace Wangshuibai. BMC Genom..

[18] Hao Y., Wang T., Kang W., Wang X., Fu Y., Huang L., Kang Z. (2016). Transcriptome Analysis Provides Insights into the Mechanisms Underlying Wheat Plant Resistance to Stripe Rust at the Adult Plant Stage. PloS ONE.

[19] Dubcovsky J., Lukaszewski A., Echaide M., Antonelli E., Porter D. (1998). Molecular characterization of two Triticum speltoides interstitial translocations carrying leaf rust and greenbug resistance genes. Crop Sci..

[20] Helguera M., Khan I.A., Dubcovsky J. (2000). Development of PCR markers for the wheat leaf rust resistance gene Lr47. Theor. Appl. Genet..

[21] Vanzetti L.S., Brevis J.C., Dubcovsky J., Helguera M. (2006). Identification of microsatellites linked to Lr47. Electron. J. Biotechnol..

[22] Brevis J.C., Chicaiza O., Khan I.A., Jackson L., Morris C.F., Dubcovsky J. (2008). Agronomic and Quality Evaluation of Common Wheat Near-Isogenic Lines Carrying the Leaf Rust Resistance Gene Lr47. Crop Sci..

[23] Gebrewahid T.W., Yao Z., Yan X.C., Gao P., Li Z. (2017). Identification of Leaf Rust Resistance Genes in Chinese Common Wheat Cultivars. Plant Dis..

[24] Wang X., Bi W., Gao J., Yu X., Wang H., Liu D. (2018). Systemic acquired resistance, *NPR1*, and pathogenesis-related genes in wheat and barley. J. Integr. Agric..

[25] Görlach J., Volrath S., Knaufbeiter G., Hengy G., Beckhove U., Kogel K.H., Oostendorp M., Staub T., Ward E., Kessmann H. (1996). Benzothiadiazole, a novel class of inducers of systemic acquired resistance, activates gene expression and disease resistance in wheat. Plant Cell.

[26] Beßer K., Jarosch B., Langen G., Kogel K.-H. (2000). Expression analysis of genes induced in barley after chemical activation reveals distinct disease resistance pathways. Mol. Plant Pathol..

[27] Li Z., Xia X., He Z., Li X., Zhang L., Wang H., Meng Q., Yang W., Li G., Liu D. (2010). Seedling and slow rusting resistance to leaf rust in Chinese wheat cultivars. Plant Dis..

[28] Li H., Mahmood T., Antony G., Lu N., Pumphreys M., Gill B., Kang Z., White F.F., Bai J. (2016). The non-host pathogen Puccinia triticina elicits an active transcriptional response in rice. Eur. J. Plant Pathol..

[29] Morant M., Bak S., Møller B.L., Werck-Reichhart D. (2003). Plant cytochromes P450: Tools for pharmacology, plant protection and phytoremediation. Curr. Opin. Biotechnol..

[30] Kotak S., Larkindale J., Lee U., von Koskull-Döring P., Vierling E., Scharf K.D. (2007). Complexity of the heat stress response in plants. Curr. Opin. Plant Biol..

[31] Mauchmani B., Mauch F. (2005). The role of abscisic acid in plant-pathogen interactions. Curr. Opin. Plant Biol..

[32] Guo J., Islam M.A., Lin H., Ji C., Duan Y., Liu P., Zeng Q., Day B., Kang Z., Guo J. (2018). Genome-Wide Identification of Cyclic Nucleotide-Gated Ion Channel Gene Family in Wheat and Functional Analyses of TaCNGC14 and TaCNGC16. Front. Plant Sci..

[33] Liu P., Duan Y., Liu C., Xue Q., Guo J., Qi T., Kang Z., Guo J. (2018). The calcium sensor TaCBL4 and its interacting protein TaCIPK5 are required for wheat resistance to stripe rust fungus. J. Exp. Bot..

[34] Steuernagel B., Witek K., Krattinger S.G., Ramirez-Gonzalez R.H., Schoonbeek H.-j., Yu G., Baggs E., Witek A., Yadav I., Krasileva K.V. (2018). Physical and transcriptional organisation of the bread wheat intracellular immune receptor repertoire. bioRxiv.

[35] Tang D., Wang G., Zhou J.-M. (2017). Receptor kinases in plant-pathogen interactions: More than pattern recognition. Plant Cell.

[36] Jones J.D., Dangl J.L. (2006). The plant immune system. Nature.

[37] Swiderski M.R., Innes R.W. (2001). The Arabidopsis PBS1 resistance gene encodes a member of a novel protein kinase subfamily. Plant J..

[38] Singh K., Foley R.C., Oñatesánchez L. (2002). Transcription factors in plant defense and stress responses. Curr. Opin. Plant Biol..

[39] Alves M.S., Dadalto S.P., Gonçalves A.B., Souza G.B.D., Barros V.A., Fietto L.G. (2013). Plant bZIP Transcription Factors Responsive to Pathogens: A Review. Int. J. Mol. Sci..

[40] Ambawat S., Sharma P., Yadav N.R., Yadav R.C. (2013). MYB transcription factor genes as regulators for plant responses: An overview. Physiol. Mol. Biol. Plants.

[41] Gao J., Bi W., Li H., Wu J., Yu X., Liu D., Wang X. (2018). WRKY Transcription Factors Associated With NPR1-Mediated Acquired Resistance in Barley Are Potential Resources to Improve Wheat Resistance to Puccinia triticina. Front. Plant Sci..

[42] Clavijo B.J., Venturini L., Schudoma C., Accinelli G.G., Kaithakottil G., Wright J., Borrill P., Kettleborough G., Heavens D., Chapman H. (2017). An improved assembly and annotation of the allohexaploid wheat genome identifies complete families of agronomic genes and provides genomic evidence for chromosomal translocations. Genome Res..

[43] Trapnell C., Pachter L., Salzberg S.L. (2009). TopHat: Discovering splice junctions with RNA-Seq. Bioinformatics.

[44] Trapnell C., Williams B.A., Pertea G., Mortazavi A., Kwan G., Van Baren M.J., Salzberg S.L., Wold B.J., Pachter L. (2010). Transcript assembly and quantification by RNA-Seq reveals unannotated transcripts and isoform switching during cell differentiation. Nat. Biotechnol..

[45] Love M.I., Huber W., Anders S. (2014). Moderated estimation of fold change and dispersion for RNA-seq data with DESeq2. Genome Biol..

[46] Young M.D., Wakefield M.J., Smyth G.K., Oshlack A. (2010). Gene ontology analysis for RNA-seq: Accounting for selection bias. Genome Biol..

[47] Keeble-Gagnère G., Rigault P., Tibbits J., Pasam R., Hayden M., Forrest K., Frenkel Z., Korol A., Huang B.E., Cavanagh C. (2018). Optical and physical mapping with local finishing enables megabase-scale resolution of agronomically important regions in the wheat genome. Genome Biol..

[48] Paolacci A.R., Tanzarella O.A., Porceddu E., Ciaffi M. (2009). Identification and validation of reference genes for quantitative RT-PCR normalization in wheat. BMC Mol. Biol..

[49] Schmittgen T.D., Livak K.J. (2008). Analyzing real-time PCR data by the comparative C T method. Nat. Protoc..

[50] Chen A., Li C., Hu W., Lau M.Y., Lin H., Rockwell N.C., Martin S.S., Jernstedt J.A., Lagarias J.C., Dubcovsky J. (2014). PHYTOCHROME C plays a major role in the acceleration of wheat flowering under long-day photoperiod. Proc. Natl. Acad. Sci. USA.

