# Supplementary material for: Genome-Wide Expression Profiling of Genes Associated with the Lr47-Mediated Wheat Resistance to Leaf Rust (Puccinia triticina)
Source: Int J Mol Sci. 2019 Sep 11;20(18):4498. doi: 10.3390/ijms20184498 (PMC6769777; doi:10.3390/ijms20184498)
Supplement: Supplementary file 1 [file ijms-20-04498-s001.zip › Supplementary Tables S1-S3.pdf]

**Supplementary Table S1.** Sequencing information of the samples in the current RNA-seq assay.

| Sample name | Raw reads | Clean reads | Clean bases | Error rate(%) | Q20(%) | Q30(%) | GC content(%) |
|---|---|---|---|---|---|---|---|
| R47_CK_1 | 139867242 | 134511230 | 20.18G | 0.02 | 96.93 | 92.27 | 56.72 |
| R47_CK_2 | 122071112 | 117533988 | 17.63G | 0.02 | 96.98 | 92.39 | 56.42 |
| R47_CK_3 | 90972864 | 87609364 | 13.14G | 0.02 | 96.85 | 92.13 | 56.45 |
| R47_PT_1 | 111161172 | 106871764 | 16.03G | 0.02 | 96.97 | 92.35 | 56.87 |
| R47_PT_2 | 94684754 | 91438232 | 13.72G | 0.02 | 96.97 | 92.36 | 57.23 |
| R47_PT_3 | 100430446 | 96508140 | 14.48G | 0.02 | 96.94 | 92.32 | 57.53 |
| R47_1037_1 | 105619412 | 105619412 | 15.84G | 0.02 | 97.06 | 92.54 | 56.86 |
| R47_1037_2 | 113428678 | 112161374 | 16.82G | 0.03 | 96.85 | 91.77 | 55.45 |
| R47_1037_3 | 120811660 | 119578618 | 17.94G | 0.03 | 96.41 | 90.78 | 55.86 |
| R47_WY_1 | 110602272 | 110602272 | 16.59G | 0.02 | 97.02 | 92.47 | 56.53 |
| R47_WY_2 | 116745456 | 115465052 | 17.32G | 0.03 | 96.58 | 91.16 | 55.63 |
| R47_WY_3 | 123319988 | 122008174 | 18.3G | 0.03 | 97.00 | 92.03 | 54.91 |

R47_CK: Seedlings of wheat line *Lr47*-Yecora Rojo-$BC_6F_5$ inoculated with sterile water.

R47_PT: Seedlings of wheat line *Lr47*-Yecora Rojo-$BC_6F_5$ inoculated with *Pt* pathotype THTT.

R47_1037: Seedlings of wheat line *Lr47*-UC1037 inoculated with *Pt* pathotype THTT.

R47_WY: Seedlings of wheat line *Lr47*-White Yecora inoculated with *Pt* pathotype THTT.

**Supplementary Table S2.** Assembly information of the samples in the current RNA-seq assay.

| **Sample name** | **R47_CK_1** | **R47_CK_2** | **R47_CK_3** | **R47_PT_1** | **R47_PT_2** | **R47_PT_3** | **R47_1037_1** | **R47_1037_2** | **R47_1037_3** | **R47_WY_1** | **R47_WY_2** | **R47_WY_3** |
|---|---|---|---|---|---|---|---|---|---|---|---|---|
| **Total reads** | 134511230 | 117533988 | 87609364 | 106871764 | 91438232 | 96508140 | 105619412 | 112161374 | 119578618 | 110602272 | 115465052 | 122008174 |
| **Total mapped** | 92.90% | 92.97% | 92.92% | 92.72% | 93.02% | 92.56% | 92.89% | 93.14% | 92.96% | 92.82% | 93% | 92.05% |
| **Multiple mapped** | 3.94% | 3.98% | 3.94% | 4.09% | 4.14% | 3.91% | 4.10% | 3.51% | 3.25% | 4.16% | 3.56% | 2.98% |
| **Uniquely mapped** | 88.96% | 89% | 88.98% | 88.63% | 88.88% | 88.65% | 88.79% | 89.63% | 89.71% | 88.66% | 89.45% | 89.07% |
| **Read-1** | 44.79% | 44.80% | 44.83% | 44.61% | 44.75% | 44.64% | 44.67% | 44.92% | 45.23% | 44.64% | 44.99% | 44.65% |
| **Read-2** | 44.17% | 44.20% | 44.15% | 44.02% | 44.13% | 44.01% | 44.12% | 44.71% | 44.48% | 44.02% | 44.46% | 44.42% |
| **Reads map to '+'** | 44.38% | 44.40% | 44.39% | 44.22% | 44.35% | 44.23% | 44.30% | 44.77% | 44.79% | 44.23% | 44.66% | 44.48% |
| **Reads map to '-'** | 44.57% | 44.60% | 44.59% | 44.41% | 44.54% | 44.42% | 44.49% | 44.86% | 44.91% | 44.43% | 44.79% | 44.59% |
| **Non-splice reads** | 57.94% | 57.93% | 57.22% | 58.01% | 57.90% | 58.61% | 58.75% | 56.70% | 58.34% | 58.46% | 56.88% | 59.71% |
| **Splice reads** | 31.02% | 31.07% | 31.76% | 30.62% | 30.99% | 30.04% | 30.03% | 32.93% | 31.36% | 30.20% | 32.57% | 29.36% |

R47_CK: Seedlings of wheat line *Lr47*-Yecora Rojo-BC6F5 inoculated with sterile water.

R47_PT: Seedlings of wheat line *Lr47*-Yecora Rojo-BC6F5 inoculated with *Pt* pathotype THTT.

R47_1037: Seedlings of wheat line *Lr47*-UC1037 inoculated with *Pt* pathotype THTT.

R47_WY: Seedlings of wheat line *Lr47*-White Yecora inoculated with *Pt* pathotype THTT.

**Supplementary Table S3.** Primers used in the current study.

| Primer name | Sequence 5' to 3' | Gene accession | PCR product | Amplification efficiency |
|---|---|---|---|---|
| LRR-RLK_AA1807990-qRT-F | GACATTTCGTCAAACAAGC | TRIAE_CS42_7AS_TGACv1_569126_AA1807990 | 88 bp | 99.0% |
| LRR-RLK_AA1807990-qRT-R | AAGCATACCAAGACCACC | | | |
| LRR-RLK_AA1807960-qRT-F | TAGGTGGGACGCTGGAGT | TRIAE_CS42_7AS_TGACv1_569126_AA1807960 | 150 bp | 112.0% |
| LRR-RLK_AA1807960-qRT-R | TTATCTGGTTGCCTTGCA | | | |
| RPM1_AA1836940-qRT-F | GACATACATGAGGTGCCTAG | TRIAE_CS42_7AS_TGACv1_570514_AA1836940 | 84 bp | 104.0% |
| RPM1_AA1836940-qRT-R | GAGTCCCTTCACATTCCA | | | |
| LRR-RLK_AA1822740-qRT-F | GGTTTGGGCTCAAGGATA | TRIAE_CS42_7AS_TGACv1_569722_AA1822740 | 81 bp | 112.0% |
| LRR-RLK_AA1822740-qRT-R | AGGACATTGCGTGGTTTC | | | |
| WAK2_AA1839280-qRT-F | TGGCCCGGTGACCACAAA | TRIAE_CS42_7AS_TGACv1_570678_AA1839280 | 155 bp | 95.9% |
| WAK2_AA1839280-qRT-R | AGAACGACACGCAGCCTCCC | | | |
| L-type RLK_AA1946750-qRT-F | CGTCGGTGGCACCACTTACA | TRIAE_CS42_7BS_TGACv1_592960_AA1946750 | 199 bp | 92.2% |
| L-type RLK_AA1946750-qRT-R | CACTGAACTTGGCTGACCAGATAGA | | | |
| ZmPK1_AA1953360-qRT-F | CAACCTTGTCGTCGTGGAT | TRIAE_CS42_7BS_TGACv1_593644_AA1953360 | 294 bp | 98.0% |
| ZmPK1_AA1953360-qRT-R | TGTTGAAGAACAGCATAGCG | | | |
| LRR-RLK_AA2043410-qRT-F | AGCAAGCATCCCAGAATC | TRIAE_CS42_7DS_TGACv1_622658_AA2043410 | 86 bp | 100.9% |
| LRR-RLK_AA2043410-qRT-R | TCATGGTAGTCAAGTCGGTTA | | | |
| TaPR1a-qRT-F | GCGGTGAAGCTGTGGGTGGA | FJ815169 | 180 bp | 94.4% |
| TaPR1a-qRT-R | GGGCTCGTAGTTGCAGGTGATGA | | | |
| TaActin-qRT-F | ACCTTCAGTTGCCCAGCAAT | AB181991 | 91 bp | 102.6% |
| TaActin-qRT-R | CAGAGTCGAGCACAATACCAGTTG | | | |
