# Supplementary material for: Genome-Wide Expression Profiling of Genes Associated with the Lr47-Mediated Wheat Resistance to Leaf Rust (Puccinia triticina)
Source: Int J Mol Sci. 2019 Sep 11;20(18):4498. doi: 10.3390/ijms20184498 (PMC6769777; doi:10.3390/ijms20184498)
Supplement: Supplementary file 1 [file ijms-20-04498-s001.zip › Supplementary Figures S1-S7.pdf]

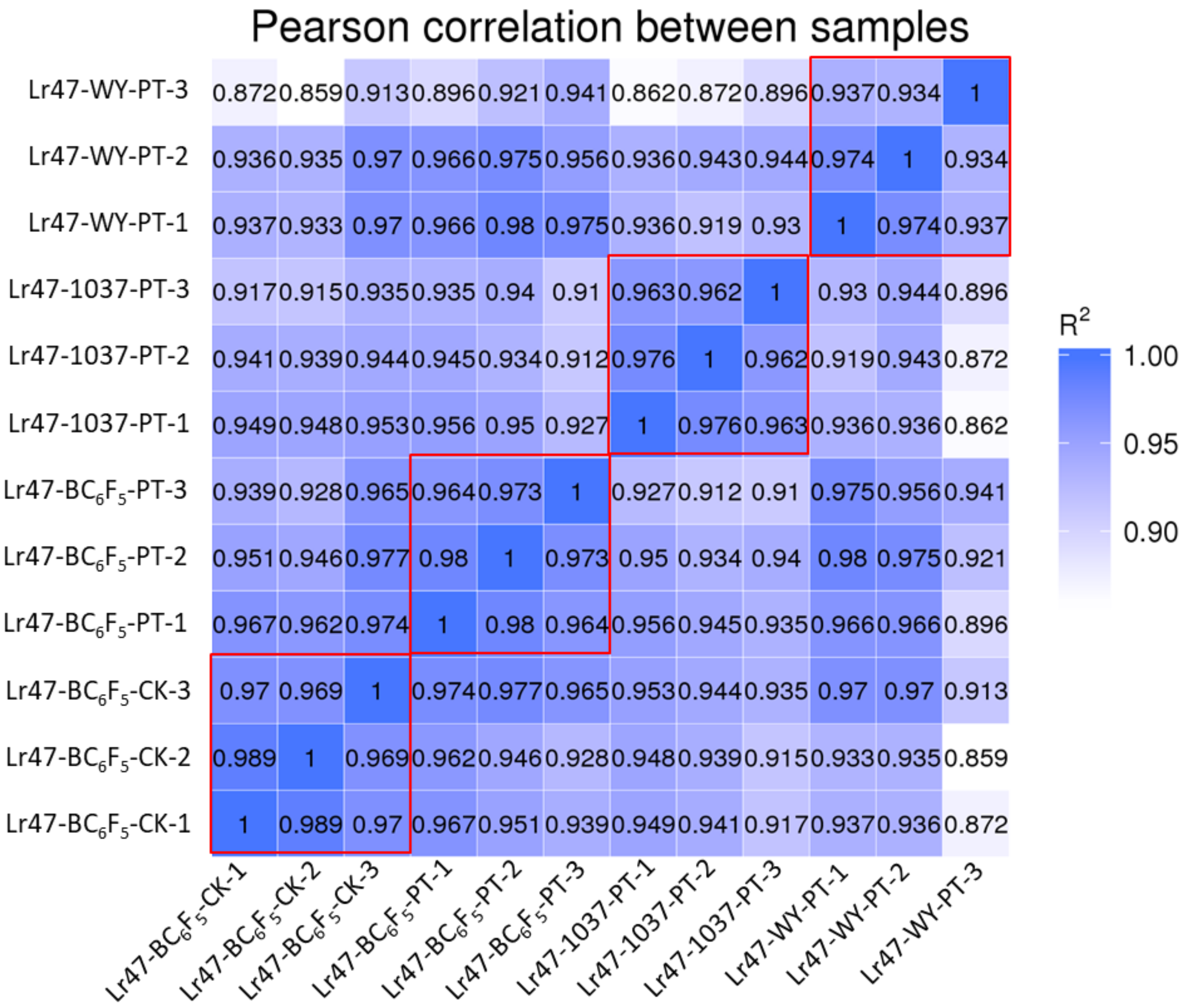

Supplementary Figure S1. Pearson's correlation of the overall gene expression levels among biological replicates in the RNA-seq assay. Three biological replicates were collected for each of the materials. Clear correlations of the overall gene expression levels between biological replicates were detected ($R^2 > 0.92$). *Lr47*-BC$_6$F$_5$-CK: Seedlings of wheat line "*Lr47*-Yecora Rojo-BC$_6$F$_5$" inoculated with sterile water. *Lr47*-BC$_6$F$_5$-PT: Seedlings of wheat line "*Lr47*-Yecora Rojo-BC$_6$F$_5$" inoculated with *Pt* race THTT. *Lr47*-1037: Seedlings of wheat line "*Lr47*-UC1037" inoculated with *Pt* race THTT. *Lr47*-WY: Seedlings of wheat line "*Lr47*-White Yecora" inoculated with *Pt* race THTT.

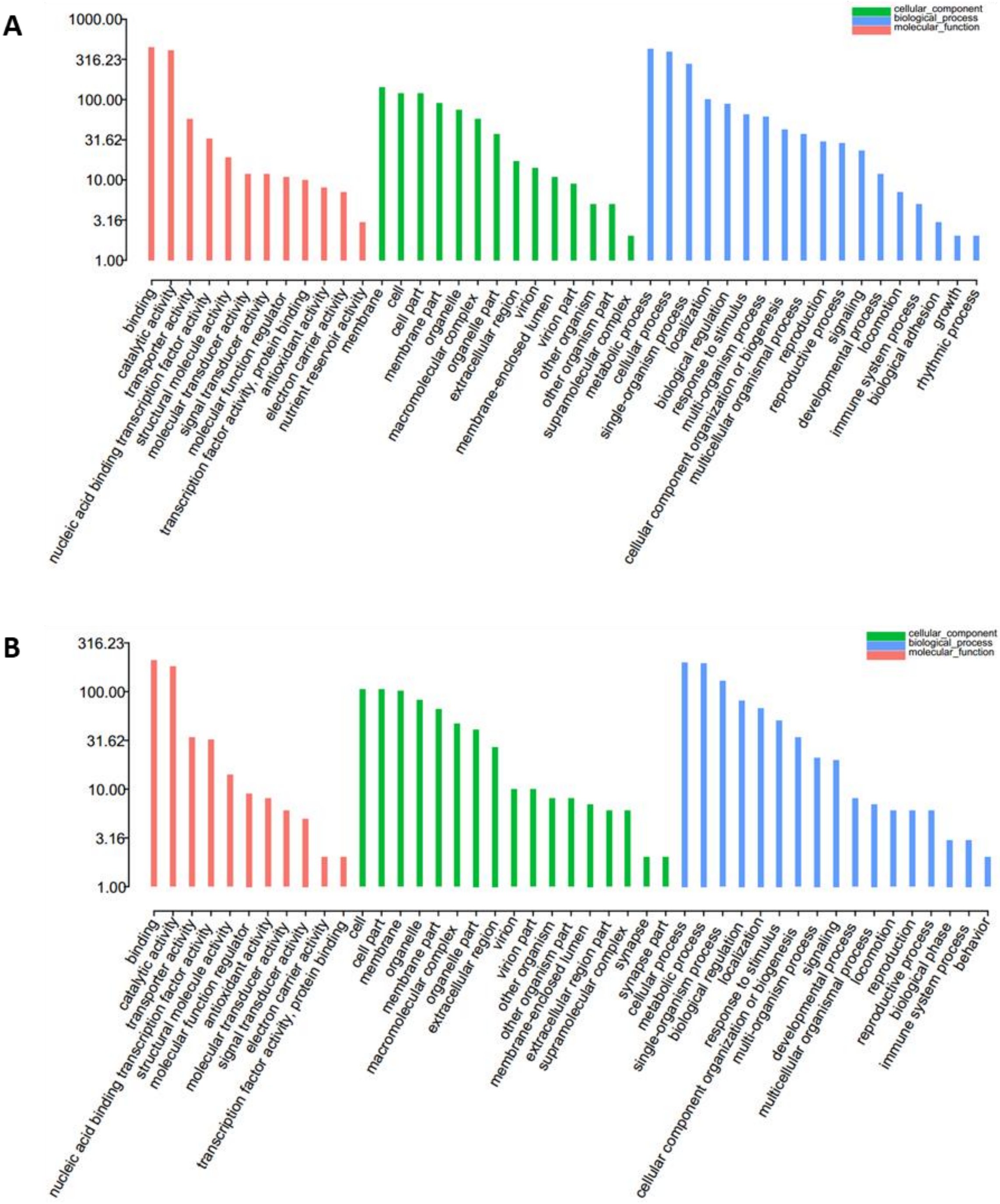

Supplementary Figure S2. GO annotations for DEGs. Compared with the mock inoculation with water, a total number of 863 upregulated (*q*-value < 0.05 and log2foldchange > 1) and 418 downregulated (*q*-value < 0.05 and log2foldchange < -1) DEGs were identified. Both upregulated (**A**) and downregulated (**B**) DEGs were categorized by their GO annotations into different functional groups of three main categories: biological process, cellular component, and molecular function. The y-axis indicates the percentage of genes in a category.

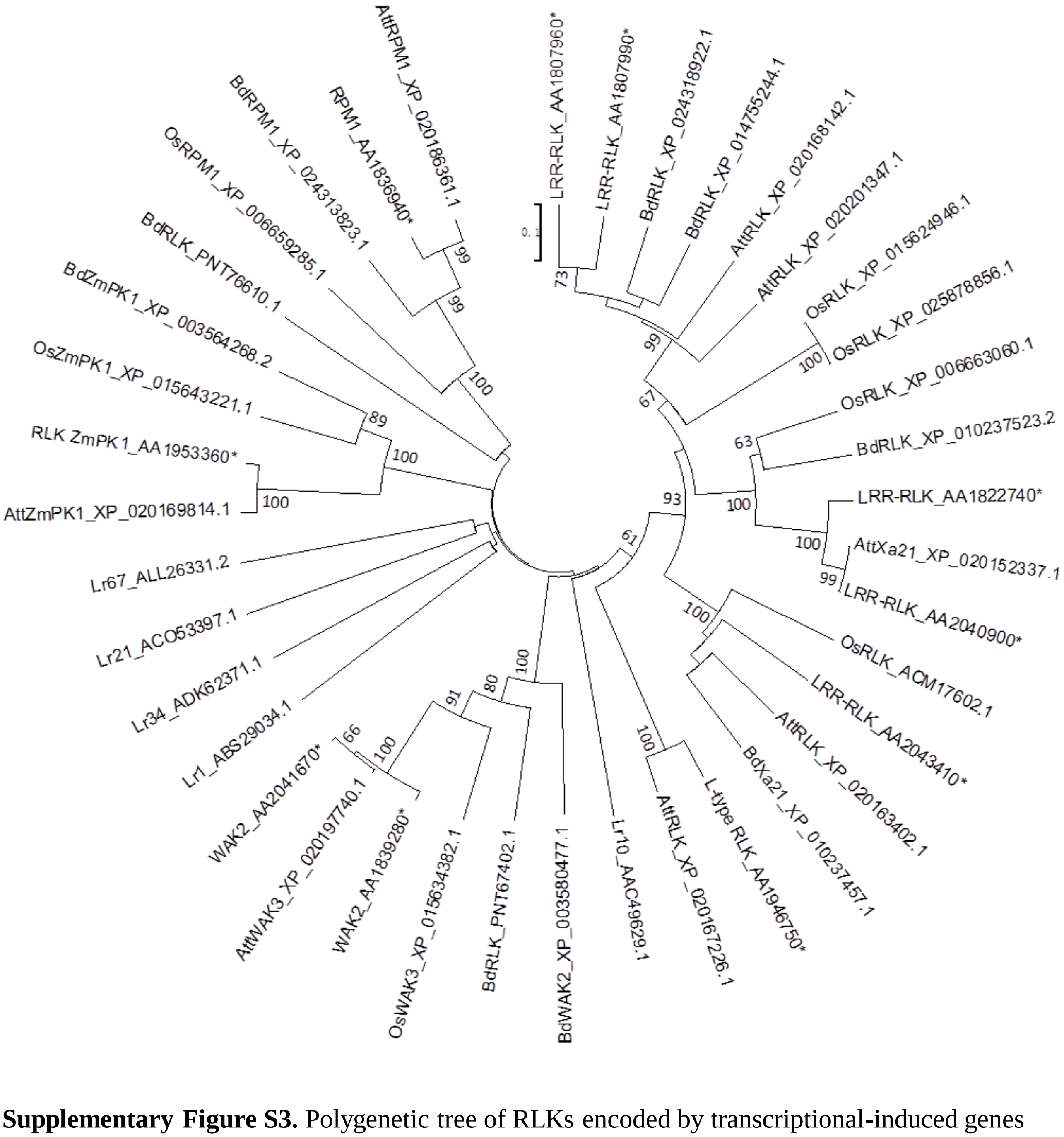

Supplementary Figure S3. Polygenetic tree of RLKs encoded by transcriptional-induced genes located on the chromosomes 7AS, 7BS, and 7DS. A neighbor-joining tree was generated by MEGA software using all ten identified RLKs from the transcriptome database and their closest homologs from *Aegilops tauschii* (Att), *Brachypodium distachyon* (Bd), and *Oryza sativa* (Os) obtained from GenBank nr database. Protein sequences deduced from the previously cloned *Lr* genes, including *Lr1*, *Lr10*, *Lr21*, *Lr34*, and *Lr67*, were also employed.

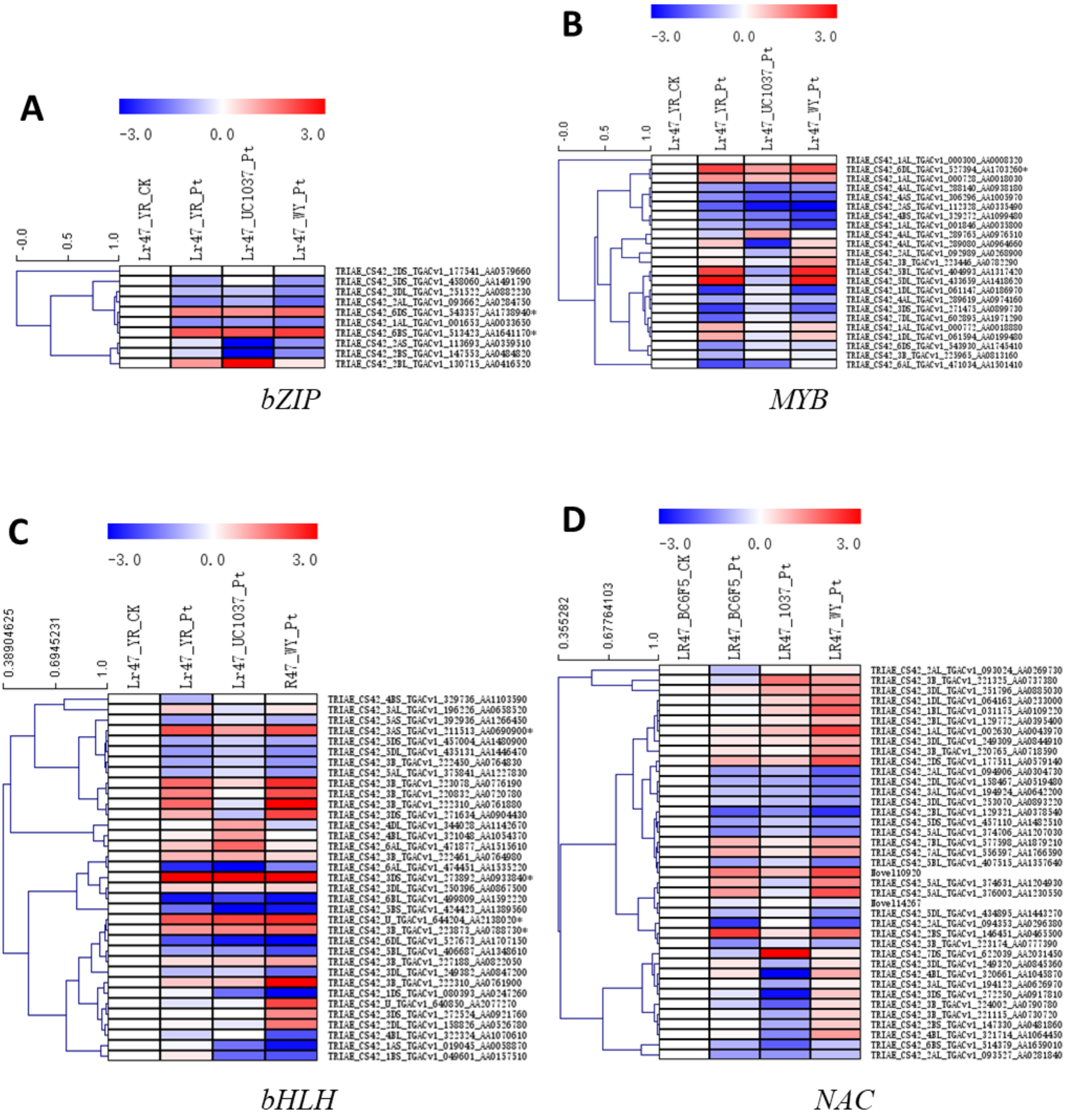

Supplementary Figure S4. Expression profiles of the genes encoding bZIP, MYB, bHLH, and NAC transcription factors during the *Lr47*-mediated resistance. FPKM values for each of the collected genes in "LR47_BC6F5_Pt", "LR47_1037_Pt", and "LR47_WY_Pt" were relative to that in "LR47_BC6F5_CK". A log2foldchange data transformation was conducted using Microsoft Excel software. Heatmaps were generated by MeV software using the relative expression data of genes encoding bZIP (**A**), MYB (**B**), bHLH (**C**), and NAC (**D**) transcription factors from the transcriptome database. Genes with similar expression patterns were clustered using the "Hierarchical Clustering" function of the MeV software. Constantly induced transcription factor genes were labeled with asterisk (*), and the deduced proteins of which were employed to generate polygenetic trees in **Supplementary Figure S7**, respectively.

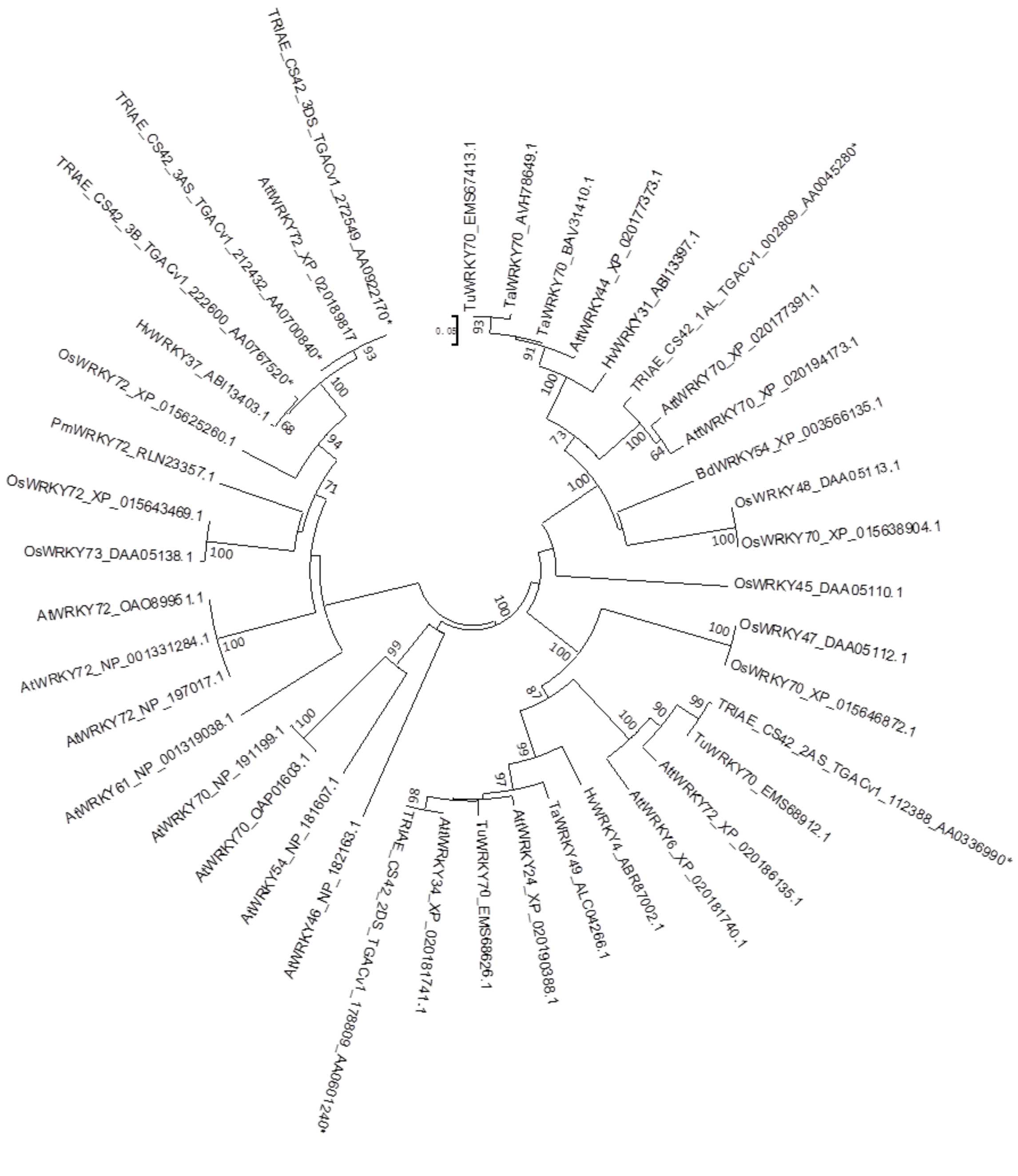

Supplementary Figure S5. Polygenetic tree of the selected WRKY transcription factor proteins. A neighbor-joining tree was generated by MEGA software using all six selected WRKY proteins from the transcriptome database and their closest homologs from *Triticum aestivum* (Ta), *Triticum urartu* (Tu), *Aegilops tauschii* (Att), *Brachypodium distachyon* (Bd), *Hordeum vulgare* (Hv), *Arabidopsis thaliana* (At), *Panicum miliaceum* (Pm), and *Oryza sativa* (Os) obtained from GenBank nr database.

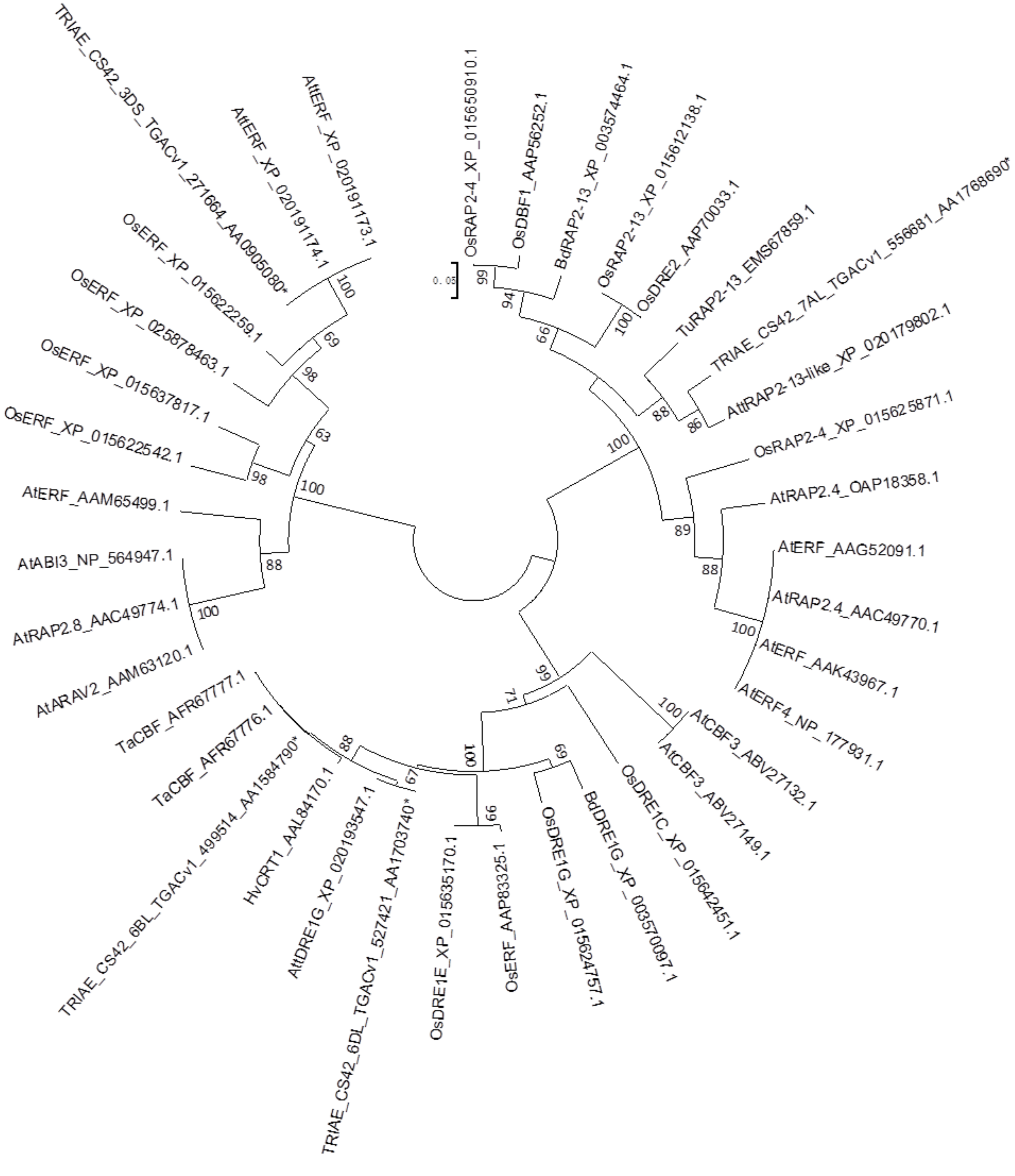

Supplementary Figure S6. Polygenetic tree of the selected ERF transcription factor proteins. A neighbor-joining tree was generated by MEGA software using all four selected ERF proteins from the transcriptome database and their closest homologs from *Triticum aestivum* (Ta), *Triticum urartu* (Tu), *Aegilops tauschii* (Att), *Brachypodium distachyon* (Bd), *Hordeum vulgare* (Hv), *Arabidopsis thaliana* (At), and *Oryza sativa* (Os) obtained from GenBank nr database.

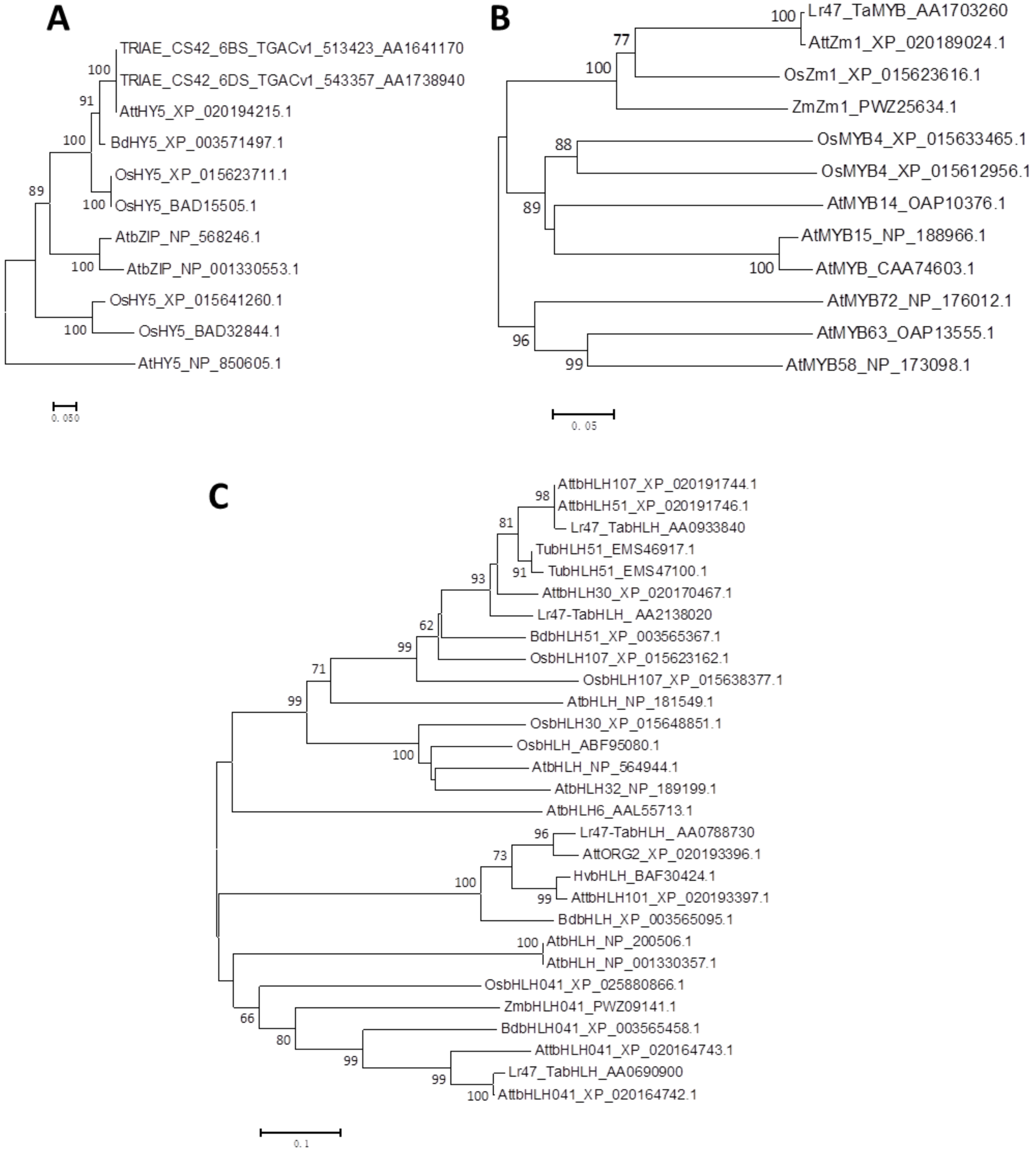

Supplementary Figure S7. Polygenetic trees of the selected bZIP, MYB, and bHLH transcription factor proteins. Neighbor-joining trees were generated by MEGA software using selected bZIP (**A**), MYB (**B**), and bHLH (**C**) proteins from the transcriptome database and their closest homologs from *Triticum aestivum* (Ta), *Triticum urartu* (Tu), *Aegilops tauschii* (Att), *Brachypodium distachyon* (Bd), *Hordeum vulgare* (Hv), *Arabidopsis thaliana* (At), *Zea mays* (Zm), and *Oryza sativa* (Os) obtained from GenBank nr database.
